# Weight Status and Dental Problems in Early Childhood: Classification Tree Analysis of a National Cohort

**DOI:** 10.3390/dj5030025

**Published:** 2017-08-31

**Authors:** Michael Crowe, Michael O’ Sullivan, Oscar Cassetti, Aifric O’ Sullivan

**Affiliations:** 1 Division of Restorative Dentistry & Periodontology, Dublin Dental University Hospital, Trinity College Dublin, Dublin, Dublin 2, Ireland; michael.osullivan@dental.tcd.ie (M.O.S.); oscar.getstring@gmail.com (O.C.); 2 UCD Institute of Food and Health, 2.05 Science Centre, South, UCD, Belfield, Dublin, Dublin 4, Ireland; aifric.osullivan@ucd.ie

**Keywords:** body mass index, diet, dental problem, classification tree

## Abstract

A poor quality diet may be a common risk factor for both obesity and dental problems such as caries. The aim of this paper is to use classification tree analysis (CTA) to identify predictors of dental problems in a nationally representative cohort of Irish pre-school children. CTA was used to classify variables and describe interactions between multiple variables including socio-demographics, dietary intake, health-related behaviour, body mass index (BMI) and a dental problem. Data were derived from the second (2010/2011) wave of the ‘Growing Up in Ireland’ study (GUI) infant cohort at 3 years, *n* = 9793. The prevalence of dental problems was 5.0% (*n* = 493). The CTA model showed a sensitivity of 67% and specificity of 58.5% and overall correctly classified 59% of children. Ethnicity was the most significant predictor of dental problems followed by longstanding illness or disability, mother’s BMI and household income. The highest prevalence of dental problems was among children who were obese or underweight with a longstanding illness and an overweight mother. Frequency of intake of some foods showed interactions with the target variable. Results from this research highlight the interconnectedness of weight status, dental problems and general health and reinforce the importance of adopting a common risk factor approach when dealing with prevention of these diseases.

## 1. Introduction

Early childhood caries (ECC) is a disease defined by the presence of one or more decayed, missing (due to caries), or filled tooth surfaces in any primary tooth in a child < 71 months old [1]. ECC is the most prevalent dental problem in pre-schoolers [2], one of the most common causes of hospital admission and the most common cause of dental extractions under general anaesthesia [1,2]. Obesity, defined as an excess of body fat [3], is another growing concern among preschool children. Body mass index (BMI) is frequently used to classify adults as overweight or obese; however, classifying overweight and obesity in children is complicated by age and gender specific differences [3,4]. For this reason, the International Obesity Task Force (IOTF) defines childhood weight status based on BMI centile curves that correspond to adult criteria from 2 to 18 years for males and females [5]. In Europe, 12%–15% of preschool children are classified as overweight or obese based on IOTF criteria [6]. Concerns around EEC and childhood obesity are heightened by the fact that both are strong predictors of these respective conditions throughout the life-course [7,8].

The preschool age is a particularly important period to minimise the risks for dental caries and obesity [9] and the primary caregiver (PCG) plays a key role in facilitating prevention through feeding patterns and other behaviours [1,8,10]. Obesity and dental caries share some common risk factors including food choice, dietary intake patterns, diet quality and socioeconomic factors such as PCG education and household income [11,12,13]. Given the associations that exist between oral health and general health interest is growing in using a common risk factor approach to investigate the multidimensional causes of dental and weight-status problems, particularly in preschool children [14,15,16,17]. Although some studies have shown a positive relationship between BMI and dental caries, others suggest that they are weakly correlated, inverse or even U-shaped and that different predictors may be associated with dental caries at both high and low BMI levels [11,12,14,17]. Indeed, very few studies report the oral health status of underweight children and often group underweight and normal weight without considering differences in risk [14,18].

Data-driven methods are being increasingly proposed to empirically derive dietary patterns associated with chronic disease [19]. Methods that aim to uncover the relationship between independent variables and a dependent variable are described as supervised learning. The discovered relationship is typically presented as a classification or regression model [20]. Thus, in Classification and Regression Tree Analysis (CART) when the target (dependent) variable is continuous a regression analysis is performed and when the target variable is categorical a classification tree analysis (CTA) is carried out. Data mining techniques are invaluable when analysing multidimensional data from large-scale survey microdata files as they provide a means to identify novel diet-disease relationships and can help establish inter-relationships between causal factors [20].

With a few exceptions, most national dental surveys tend to focus on children aged 5 and older. While nationally representative studies of obesity prevalence in older Irish children are well documented [21] there are few, apart from a National Preschool Nutrition Survey [22] that relate to pre-schoolers. The research in this secondary analysis proposed to use a flexible analytical approach (CTA) to explore the multilevel relations between weight status and dental problems in a large, nationally representative cohort of 3-year old children from the ‘Growing Up in Ireland’ study (GUI).

## 2. Methods

### 2.1. Data Collection and Participants

The aim of the GUI infant survey is to determine the individual, family and wider social and environmental factors that affect the development of children. GUI is a nationally representative longitudinal study that collected data from infants at 9 months (Wave-1) and followed up when children were 3-years old (Wave-2) providing the data file used in this study with 9793 cases. Between December 2007 and June 2008 GUI selected a random sample on a systematic basis, pre-stratified by marital status, county of residence, nationality and number of children from the National Child Benefits Register which is a universal welfare entitlement in the Republic of Ireland [23]. The sampling fraction was 0.42 with an overall response of 64.5% providing 11,134 families in Wave 1. Follow-up interviews for Wave-2 occurred between December 2010 and July 2011 and 91% of families responded while 3.8% emigrated or deceased and the remainder either refused or were not contactable. The PCG was interviewed in the family home using a questionnaire after written informed consent was obtained [23].

### 2.2. Anthropometric Measurements

A standard (Leicester) portable height stick was used to measure height of PCGs and children. The weight of the children was recorded using a digital scales (SECA 835, Hamburg, Germany). Data for height and weight of the PCGs were used from Wave-1 measurements and only taken at Wave-2 if they were missing or required rechecking. PCG weight was recorded using a flat mechanical scale (SECA 761, Hamburg, Germany).

BMI was calculated as weight divided by height squared (kg/m^2^) and, for children, classified as overweight, obese, normal weight or thinness according to the IOTF age and gender specific cut-offs for 3-year olds [5,24]. For simplicity, the classification of thinness (low BMI for age) is also described as underweight in this paper although the latter strictly means low weight for age in children. Overweight and obesity for children was also classified using the UK adaptation of percentile cut-offs from the WHO Multicentre Growth Reference Study (MGRS) with overweight criteria defined as a BMI between the 91st and 98th percentile while obesity was defined as a BMI on or greater than the 98th percentile [25]. PCG BMI was categorised into underweight (BMI < 18.5), normal (BMI 18.5–24.9), overweight (BMI 25–29.9) and obese (BMI > 30).

### 2.3. CTA Target Variable

The dichotomous target variable was a PCG reported dental problem. The question asked was: Has <child> been to visit the dentist because of a problem with his/her teeth?

### 2.4. CTA Predictor Variables

Attributes (independent variables) that were relevant to the target variable (dependent variable) were selected for inclusion in the model based on findings from previous research. The demographic and socioeconomic variables selected were child gender, PCG age and gender, ethnicity, PCG education level, family social class and annual equivalised household income [1,14,15,16,26,27]. Ethnicity was defined as Irish, Any other White background, Black, Asian or Other. The highest education level attained by the PCG was one of thirteen categories ranging from no formal education to doctorate level which was collapsed to five groups for descriptive analysis. Family social class was measured using the Irish Central Statistics Office’s classification based on occupation, categorising families into one of seven groups which was collapsed to four groups for descriptive analysis. Annual disposable household income was calculated by using an equivalence scale to “weight” each household for differences in size and composition with respect to number of adults and children [23]. Markers of health status [12,13,26,28] included PCG reported child illness, disability, allergies and injuries, as well as TV-viewing hours, tooth-brushing, soother/thumb-sucking, and breastfeeding as markers of health behaviour [9,14,26,29]. Dietary intake [9,11,26] was assessed using a modified version of the Sallis-Amherst Food Frequency Questionnaire from the Longitudinal Study of Australian Children (LSAC) [30]. PCG reported the child’s frequency of consumption of 15 food categories (e.g., ‘sweets’, ‘fizzy drinks/minerals/cordials’) over the previous 24-h as once, more than once or none at all.

### 2.5. Data Analysis

Wave-1 GUI data were statistically re-weighted to represent the population. Wave-2 data was weighted for attrition between waves and emigration combined with the Wave-1 weight [31].

Classification tree analysis (CTA) is based on recursive partitioning whereby the algorithm repeatedly creates splits in the sample based on the most significant predictor variable. The root node contains the entire sample and each subsequent split results in child nodes with the proportion of the classes displayed together with an adjusted P value. For this analysis the following parameters were selected in either SPSS (v. 20.0: SPSS, Chicago, IL, USA) or SPSS modeller (IBM SPSS Modeler v. 14.2: Chicago, IL, USA) using the Chi-squared Automatic Interaction Detection (CHAID) algorithm [32]: maximum tree depth = 5, parent node = 100, child node = 50 and bonferroni-adjusted chi-square statistic, significance < 0.05. A 10-fold cross-validation assessed model performance and produced an average misclassification risk. Details of the analysis methods were previously reported [33]. The degree of missing cases in the 3-year old GUI infant cohort was small except for the PCG BMI (5.2%), equivalised annual income (5.5%) and child BMI (2.6%), as previously reported [29]. The CHAID algorithm handles missing values by defining a separate category and treating them as a single category so that they are not excluded in the analysis [34]. A binary logistical regression analysis (*forward-wald*) was also conducted to compare findings with those generated by the classification tree output. A confusion matrix for a binary classifier provided estimation of selected performance metrics.

## 3. Results

### 3.1. Cohort Profile

Five percent of 3-year olds had a dental problem. As is common in investigations of health outcome the class distribution of the dataset was imbalanced. The minority class was the positive instances of having ‘a dental problem’ and the negative response was the majority class.

Table 1 describes the cohort characteristics, including anthropometric measurements, child health and behaviours. Almost all of the self-identified PCGs were female and the biological parent of the study child. Eighty five percent were ‘Irish’. Using the IOTF cut-offs [5] the prevalence of thinness and obesity were 5.7% each with an additional ~18% of children being overweight. Using the WHO growth charts and BMI cut-offs, the prevalence of overweight was 18.5% and obesity was 12.8%.

**Figure 1:**
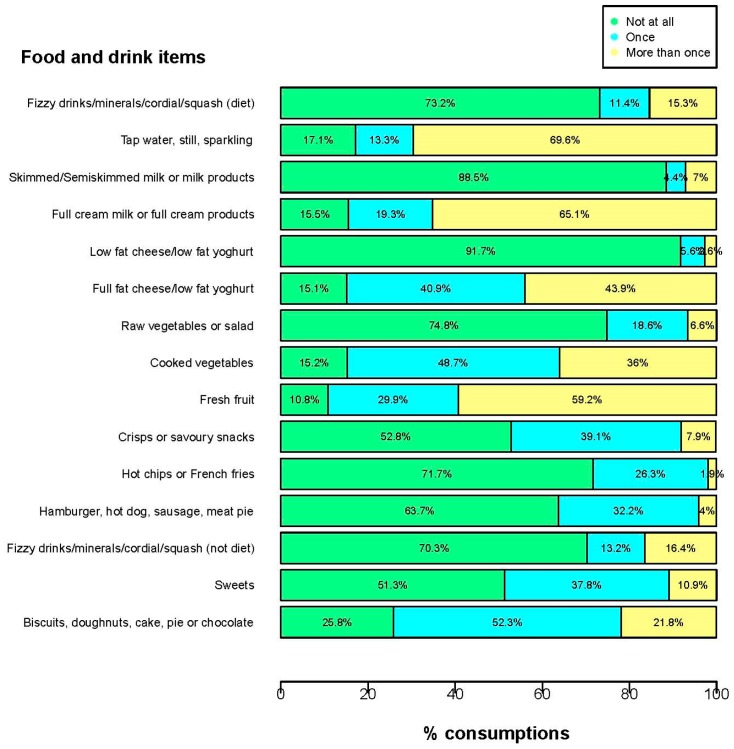
Food and drink items consumed in previous 24 h by the Growing Up in Ireland infant cohort at 3-years of age.

The frequency of food items consumed are reported in Figure 1. The majority of children consumed water’ (~83.0%), ‘full-fat milk/cream’ (~84.5%), ‘full-fat cheese/yoghurt’ (~85.0%), ‘cooked veg’ (~85.0%), ‘fresh fruit’ (~89%), and ‘biscuits/doughnuts/cake/chocolate’ (~74%) once or more than once in the previous 24-h. Of interest, a considerable proportion of 3-year olds consumed “un-healthy” foods including ‘crisps’ (~47%), “hot-chips” (~28%), sugar containing drinks (~30%), and sweets (~49%).

### 3.2. Classification Tree Analysis

**Figure 2:**
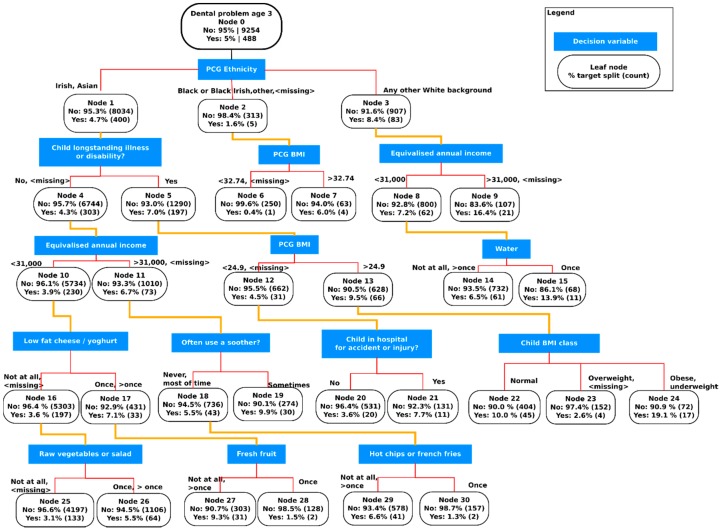
Prevalence of reported dental problems by the Growing Up in Ireland infant cohort at 3-years of age among classification tree subgroups, percentage (%) and number (*n*) in each class.

CHAID analysis generated a CTA output as depicted in Figure 2 with 30 nodes, including 17 terminal nodes. Each node contains the number and percentage of infants in each category for the dependant variable (dental problem), the categories chosen by CHAID for the predictor variable and the cut-off points for continuous variables. PCG ethnicity was the most important predictor of the 3-year old child having a dental problem splitting the root node. Twelve predictor variables were included in the final tree (Bonferroni-adjusted *p* < 0.05). Two predictors appeared twice in the output, PCG BMI (nodes 2 and 5) and equivalised household annual income (nodes 3 and 4). A confusion matrix (Table 2) produced performance metrics for the classification tree: sensitivity 66.8%, specificity 58.5% and overall accuracy 58.9%.

The ethnic subgroups were split into 3 nodes with the highest prevalence of dental problems (8.4%) among those children from a “non-Irish white” background (Node-3). Node-1 contained almost 87% of the sample (Irish and Asian ethnicity) with a 4.7% prevalence of dental problems. The tree output from node-1 to nodes 22–24 delineated subgroups linking child BMI categories with dental problems by the following predictors: PCG from an Irish/Asian background (node-1), the presence of a longstanding illness or disability in the child (node-5) and an overweight mother (node-13). The final predictor at node-13 was BMI classification of the child which split into three terminal nodes resulting in normal, overweight/missing and obese/underweight subgroups. The highest dental problem prevalence (19%, *n* = 17) was in those children in this final subgroup who were obese or underweight (node-24). Also, the subgroup at node-1 who had a longstanding illness or disability had a reported dental problem prevalence of 7.0% while those with no illness or disability had a prevalence of 4.3% (node-4). The food variables included in the tree output were ‘water’ (level-3), ‘low-fat cheese/yoghurt’ (level-4) and ‘raw vegetables/salad’, ‘fresh fruit’ and ‘hot chips’.

Logistic regression failed to generate a significant model (chi-square (6) = 9.38, *p* = 0.15).

## 4. Discussion

This study used a CHAID classification tree as a method to classify the dataset and identify relationships between the predictor variables selected and the target variable (a PCG-reported dental problem requiring a visit to the dentist). PCG ethnicity was the most significant predictor of dental problems in the CTA model and the highest prevalence of dental problems in this study was among children who were obese or underweight with a longstanding illness and an overweight PCG.

This analysis was carried out using data from a nationally representative cohort of 3-year old children, the largest child population study ever carried out in Ireland, which includes a wide range of PCG and child health and development characteristics. CTA is a non-parametric method which handles nominal and numeric input and classification trees are ideal for representing complex interactions [20]. The output produces a visualisation of all the significant interactions with the target variable at multiple levels and can, potentially, uncover subgroups that might not be discovered using other data methods [34]. The partitioning variable at the first level of the classification tree was PCG ethnicity while at the next level the most significant predictors of dental problems were the child having a longstanding illness, PCG BMI and household income. It has been reported that trends in overweight and obesity differ among different ethnic groups, even at the early preschool age, and that this cannot be explained by variations in household income [35]. Similarly, the disparities in dental caries prevalence between different ethnic groups is not fully explained by social inequalities [27]. Surprisingly, ethnicity has been used as a variable in relatively few studies of dental caries in children while the PCG education level, although it was not a significant predictor in our CTA model, has been consistently shown to be an important risk factor for caries in children [26].

While the results of our CTA are exploratory they identify certain characteristics previously suggested as risk factors or risk indicators for both obesity and dental caries [14,26,36]. The study supports recent views that data driven-outcome dependant methods such as CTA are potentially useful for investigating dietary components or patterns most associated with a health outcome and are a valid, non-parametric, alternative to logistic regression analysis [19]. The results of this analysis must be cautiously interpreted by gauging the model performance (Table 2) and understanding the limitations of both the data structure and classification tree algorithms. An imbalanced class distribution has been characterised as “one that has many more instances of some classes than others [37]. CTA of imbalanced data sets tends to result in high predictive accuracy for the majority class and low accuracy for the minority class [34]. In most health outcome investigations, including dental problems, the correct classification of the minority class is of greater interest or value than that of the majority class. The confusion matrix (Table 2) shows the results of the actual and predicted classifications carried out by CTA. The metrics calculated include sensitivity or recall (66.8%) which is the proportion of actual positive cases correctly predicted by the model and specificity (58.5%) which is the proportion of actual negative cases correctly identified by the model. The overall accuracy (58.9%) indicated the proportion of the total number of correct predictions. Logistic regression did not perform well as a classifier and none of the same input variables were significant in the final regression model. This may be due to inherent differences in the way CTA captures the division of the classes by partitioning the space using multiple decision boundaries whereas logistic regression uses a single linear decision boundary [34]. To be suitable as a prediction model for targeting risk it has been suggested that both sensitivity and specificity should be 80% or the sum be at least 160% [38].

While overweight and obesity dominate the focus of recent research with children, it is important to consider underweight (thinness) in early childhood as a condition related to poor health outcomes also. There is some evidence to suggest that dental caries may be associated with children who are underweight and suffer with slow growth due to pain on mastication [15,18,28]. The results (Table 1) shows a similar prevalence of underweight and obese children. A small subgroup (node-24) of children which combined obese and underweight categories had the highest prevalence of dental problems (19%) in the sample. This group were predominantly Irish with a longstanding illness and had an overweight PCG. It should also be noted that normal weight children (node-22) in this group had a dental problem prevalence of 10% approximately half that of the obese/underweight group, but double that of the overall sample. This finding of itself highlights the interconnectedness of weight status, dental problems and general health and reinforces the importance of adopting a common risk factor approach when dealing with prevention of these diseases [13,39]. However, while of interest in classifying this dataset, it is important to be cautious when interpreting these subgroups identified by CTA as hierarchical splitting means that they are mutually exclusive. Furthermore, successful targeting of high risk population subgroups for problems with both weight status and dental health would require a risk prediction model with both high sensitivity and specificity.

The prevalence of PCG-reported dental problems requiring a visit to the dentist was 5% which may be an under-estimate given that dental problems are often not treated unless symptomatic in the preschool years [28]. This age is a pivotal period for development of both obesity and dental caries as patterns of eating behaviour that predispose to later development of these conditions are established [8,10,15]. The prevalence of overweight or obesity in 3-year old children determined by IOTF cut-offs was approximately 23% which was similar to previous reports [22]. Almost 47% of PCG’s were overweight or obese and it is well established that parental overweight and obesity increases the risk of a child becoming overweight [39]. There are limitations in using BMI as an indirect measure of “fatness” particularly with respect to children [4] and it is important to note that there is no reference population in Ireland for grading BMI. In the CTA, we used the IOTF classification as the more conservative estimate of obese children with a higher cut-off threshold. The FFQ adopted for the GUI survey was a modified dietary screening recall and provided an indication of types and frequency of foods consumed. While PCG-reported measures of foods consumed on a single occasion may be useful in differentiating patterns of food intake it does not provide a good estimate of usual daily consumption and cannot accurately capture total energy or total nutrient intake [40]. Preschool children with unhealthy eating habits have an increased likelihood of experiencing dental caries [10]. While obesity and dental caries are both diet-mediated diseases it is clear that sugars are required in the diet for dental caries to occur [41] whereas a high consumption of energy dense foods including sugars and saturated fats are linked with obesity [16,39]. Fundamentally, obesity occurs due to an energy imbalance between calories consumed versus those expended over a period of time [11,39]. Approximately 74% of the children in GUI consumed biscuits, doughnuts, cake, pie or chocolate at least once or more than once in a 24-h period (Figure 1). Almost 49% of children ate sweets and 30% drank non-diet fizzy drinks, minerals, cordials or squash at least once or more than once. Dietary interventions aimed at reducing the intake of these unhealthy food groups may help impact on both obesity and dental caries but further investigation of these factors in longitudinal studies is still required, especially given the temporal and cumulative aspects of both conditions.

The results of this data analysis may help raise awareness among clinicians and nutrition researchers of the interrelations between weight status and dental problems, even before the primary dentition is complete. Classification tree analysis visually demonstrates how factors, such as the BMI of the primary caregiver (PCG), can interact at multiple levels and affect different subgroups of the child population. Future intervention strategies for oral health should involve consideration of the weight of both the young child and PCG at both the patient and population level. This approach has been advocated recently as both obesity and dental caries may be more likely to occur in the same populations [17]. Given the increasing public health implications of these conditions adopting a more interdisciplinary approach to shared risk factors may assist in the reduction of both problems.

A limitation of CTA is that the hierarchical nature of recursive partitioning has an inherent instability to small changes in the learning data [34]. Overfitting of the model is a known problem and this can be guarded against by using cross-validation which splits the dataset into a training portion and test portion before estimating an average misclassification risk [20]. While dental caries is the most common dental problem at this age the survey did not include a report of the outcome of the dental visit. However, it has been shown that dental disease and treatment need of young children are associated with parents’ perceptions of their children’s oral health status [42]. Also, it is important to note that this study was based on a cross sectional rather than longitudinal analysis of the infant cohort from the GUI survey. The data is reliant on PCG reporting and this is subject to recall bias and social desirability, particularly in relation to reporting of food and drink perceived as ‘healthy’ or ‘unhealthy’. Further research is focussed on maximising the quality of food intake data by augmenting the GUI survey data with more reliable dietary intake values from a national nutritional database [43] and using the longitudinal aspect of the next wave of the GUI infant cohort. Inclusion of more detailed oral health measures should also be considered.

## 5. Conclusions

The highest prevalence of dental problems in this study was among children who were obese or underweight with a longstanding illness and an overweight PCG. Societal changes may require renewed focus on oral health policies to focus on minority groups and CTA is a novel approach for exploring large survey data and health-related outcomes. The common risk factor approach may be a pragmatic means of developing shared modifiable strategies for prevention of both dental and weight problems.

## Figures and Tables

**Table 1:** Weighted ^a^ Sample Characteristics, Growing Up in Ireland infant cohort participants 2010/11 (Child 3-years of age).

	Child		PCG	
			Mean	SD
**Age (years)**			29.6	(6.1)
**Gender**	***n***	**%**	***n***	**%**
Male	5024	51.3	161	1.6
Female	4769	48.7	9632	98.4
**Anthropometrics**	**Mean**	**SD**	**Mean**	**SD**
Weight (Kg)	15.27	(2.02)		
Height (m)	95.48	(3.92)		
Body Mass Index (Kg/m^2^)				
Total	16.71	(1.61)	25.99	(5.16)
Male	16.99	(1.52)	26.98	(5.59)
Female	16.71	(1.61)	25.97	(5.15)
**BMI Categories**	***n***	**%**	***n***	**%**
Thinness IOTF	557	5.7	166	1.7
Normal IOTF	6685	68.3		
Normal WHO	6464	66.0	4523	46.2
Overweight IOTF	1737	17.7		
Overweight WHO	1815	18.5	2941	30.0
Obese IOTF	559	5.7		
Obese WHO	1257	12.8	1655	16.9
Missing	256	2.6	508	5.2
**Child Health and Behaviours**	***n***	**%**		
Dental Problems (in last 12 months)	493	5		
Longstanding illness or disability	1543	15.8		
Hospital admission (ever)	1569	16.1		
Tooth brushing 2 or more per day	5107	52.2		
Tooth brushing <2 per day	4685	47.8		
Thumb sucking	765	7.8		
Soother	3163	32.3		
TV viewing time (min/day)	1133	(72.0)		
TV viewing 1 hour or less per day	3569	36.4		
TV viewing 2 hours or less per day	3587	36.6		
TV viewing 2 hours or more per day	2630	26.9		
**Socio-Demographics**			***n***	**%**
Ethnicity				
Irish			8261	84.4
Non-Irish white			1018	10.4
Black			252	2.6
Asian			202	2.1
Other			54	0.6
**Family Social Class**				
Professional/Managerial			4553	46.5
Other non-manual/Skilled manual			3233	33.0
Semi-skilled/Unskilled			1061	10.8
Unclassified			947	9.7
**Highest Education Level**				
Lower secondary or less			1361	13.9
Upper secondary			3192	32.6
Non-degree			2080	21.2
Third level			3144	32.1
			**Mean**	**SD**
Equivalised Annual Income (€)			18,004	(10,997)

Data presented as mean and standard deviation (SD) or n and percentage. ^a^ Sample weighting factors applied to statistically adjust the data to be more representative of the population. IOTF, International Obesity Task Force; WHO, World Health Organisation.

**Table 2:** Confusion matrix showing selected performance measures for Classification tree analysis of dental problem prevalence in the Growing Up in Ireland infant cohort at 3-years of age.

Observed		Predicted Dental Problem		Percentage Correct	Measure
		Yes	No		
Dental problem	Yes	326	162	66.8%	Sensitivity ^a^
	No	3839	5415	58.5%	Specificity ^b^
Overall percentage				58.9%	Accuracy ^c^

^a^ Sensitivity = True Positive Rate = Number of True Positives/(Number of True Positives + Number of False Negatives); ^b^ Specificity = True Negative Rate = Number of True Negatives/(Number of True Negatives + Number of False Positives); ^c^ Accuracy = (True Positives + True Negatives)/(True Positives + False Positives + False Negatives + True Negatives)
